# Molecular analysis of OXA-48-producing *Escherichia coli* in Switzerland from 2019 to 2020

**DOI:** 10.1007/s10096-022-04493-6

**Published:** 2022-09-14

**Authors:** Jacqueline Findlay, Vincent Perreten, Laurent Poirel, Patrice Nordmann

**Affiliations:** 1 Medical and Molecular Microbiology, Department of Medicine, Faculty of Science and Medicine, University of Fribourg, Chemin du Musée 18, Fribourg, Switzerland; 2 Division of Molecular Bacterial Epidemiology & Infectious Diseases, Institute of Veterinary Bacteriology, University of Bern, Bern, Switzerland; 3 Swiss National Reference Center for Emerging Antibiotic Resistance (NARA), University of Fribourg, Fribourg, Switzerland; 4 INSERM European Unit (IAME, France), University of Fribourg, Fribourg, Switzerland; 5 Institute for Microbiology, University of Lausanne and University Hospital Centre, Lausanne, Switzerland

**Keywords:** Escherichia coli, OXA-48, Carbapenemase, Plasmid, Epidemiology

## Abstract

OXA-48-type ß-lactamases are the most prevalent carbapenemase-type in Enterobacterales in Switzerland, predominantly found in *Escherichia coli* and *Klebsiella pneumoniae*. Bacteria-producing OXA-48-type enzymes are endemic in some parts of the world, including Europe and North Africa, and are a frequent cause of nosocomial infections. Despite the emergence of numerous OXA-48-type variants, the original variant, OXA-48, remains the most prevalent in *E. coli*. This study describes the epidemiology of OXA-48-producing *E. coli* isolates submitted to the Swiss National Reference Center for Emerging Antibiotic Resistance (NARA) between January 2019 and December 2020.

**Supplementary Information:**

The online version contains supplementary material available at 10.1007/s10096-022-04493-6.

## Introduction

*Escherichia coli* is one of the most frequent causes of infections worldwide, particularly infections of the urinary tract, and the increasing global incidence of multidrug-resistant *E. coli*, including those that produce carbapenemases, presents a major public health threat [1]. OXA-48-type carbapenemases were first described in a Turkish carbapenem-resistant *Klebsiella pneumoniae* isolate identified in Paris in 2004 [2] and have since gone on to be reported globally, predominantly found in Enterobacterales [3]. Bacteria-producing OXA-48-type enzymes are endemic in some parts of the world, including Europe, the Indian subcontinent, and North Africa, and are a frequent cause of nosocomial infections [3, 4]. To date, forty-five OXA-48-type variants have been identified [5], although fourteen of these have been found in the chromosome of *Shewanella* species (water-borne Gram-negative species), the origin of the OXA-48-type enzymes, and the remaining 31 variants have been found in Enterobacterales species [5]. OXA-48-type enzymes typically possess activity against penicillins, narrow spectrum cephalosporins, and weakly hydrolyse carbapenems, often resulting in low carbapenem MICs and subsequently difficulties in their detection [6]. Additionally, some variants, such as OXA-163 and OXA-405, possess almost no carbapenemase activity and instead exhibit ESBL properties [7, 8]. In Switzerland, OXA-48-type enzymes are the most prevalent carbapenemase type, predominantly found in *E. coli* and *K. pneumoniae* [9]. The successful proliferation of OXA-48-type enzymes has been attributed both to successful clones, including those with chromosomally encoded *bla*_OXA-48_ [10] and to plasmid spread, particularly of the highly transferable ~ 62-kb IncL plasmid, pOXA-48a [11]. Despite the emergence of numerous OXA-48-type variants, the original OXA-48 remains the most prevalent in *E. coli* globally, although this can differ by geographical region [3]. Since 2017, carbapenemase producers are notifiable in Switzerland and are sent to the Swiss National Reference Center for Emerging Antibiotic Resistance (NARA) for further characterization. Between January 2019 and December 2020, NARA received 143 *E. coli* isolates producing OXA-48-type enzymes, of which 60 produced OXA-48, 44 OXA-244, 38 OXA-181, and one OXA-484. A recent report in Switzerland has already described the dissemination of *E. coli* isolates producing the chromosomally encoded OXA-244, a single point mutant of OXA-48 [12], predominantly corresponding to a successful strain background, namely, Sequence Type 38 [13].

In this study, we investigated the molecular characteristics and epidemiology of the 60 OXA-48-producing *E. coli* isolates submitted to NARA during this period.

## Materials and methods

### Bacterial isolates, identification, and susceptibility testing

Isolates were submitted to the NARA reference laboratory from hospitals and clinics throughout Switzerland, over a 2-year period, from January 2019 to December 2020. Patient and isolation source data was obtained from the accompanying request forms sent by referring laboratories. Species identification was confirmed using API-20E tests (bioMérieux, https://www.biomerieux.com) and UriSelect 4 agar (Bio-Rad, https://www.bio-rad.com). Susceptibility testing was performed by disk diffusion and results were interpreted in accordance with EUCAST guidelines [14]. All isolates were subjected to the detection of carbapenemase activity by the Rapidec Carba NP test (bioMérieux) and then to NG-Test CARBA 5 test (NG Biotech), according to the manufacturer’s instructions. OXA-48-type alleles were confirmed by PCR [15] and subsequent Sanger sequencing.

### Conjugation experiments

Conjugation assays were performed as follows: overnight broth cultures of the sodium azide–resistant recipient strain *E. coli* J53 and donor strains were mixed in a 4:1 ratio, and cells were collected by centrifugation and resuspended in 30 µL of cold saline. Five-microliter aliquots of the resuspension were spotted onto LB agar plates and incubated at 37 °C for 6 h. Growth was collected and resuspended in cold saline and inoculated onto LB agar plates containing temocillin (OXA-48 producers being temocillin resistant) at 50 mg/L and sodium azide at 100 mg/L. Transconjugants were confirmed by susceptibly testing, PCR amplification, and subsequent sequencing of the *bla*_OXA-48_ gene.

### Whole-genome sequencing and analyses

Whole-genome sequencing (WGS) was performed on a subset of 55, randomly selected, isolates on a NovaSeq 6000 instrument (Illumina) using the Nextera library preparation method with 2 × 150 bp paired end reads. Reads were assembled into contigs using the Shovill pipeline (https://github.com/tseemann/shovill). Sequence types, the presence of resistance genes, and speciation were confirmed, using MLST version 2.0, ResFinder version 4.1 [16], and KmerFinder version 3.2 on the Center for Genomic Epidemiology platform (https://cge.cbs.dtu.dk); contigs were annotated using Prokka [17]. Phylotypes were assigned using EzClermont (https://ezclermont.hutton.ac.uk). A core genome single-nucleotide polymorphism (SNP) alignment was generated using Parsnp [18] and viewed using Interactive Tree of Life version 6.1.1 using *E. coli* MG1655 (GenBank accession no. NC_000913) as the reference sequence.

For long read sequencing, total genomic DNA (gDNA) of isolates was extracted from a bacterial culture grown overnight using the QIAamp DNA Mini Kit (Qiagen, Valencia, CA, USA) and sequenced using the MinION Mk1C (Oxford Nanopore Technologies, Oxford, UK). Sequencing libraries were prepared using a native barcoding kit (EXP-NBD104; Oxford Nanopore Technologies, UK) and 1D chemistry Ligation Sequencing Kit (SQK-LSK109; Oxford Nanopore Technologies) and performed on a R9.4.1 Flow Cell (FLO-MIN106; Oxford Nanopore Technologies). Hybrid assembles, using both short and long-read data, were performed using UniCycler [19].

Reads were mapped to reference sequences using CLC Genomics Workbench (QIAGEN, https://www.qiagen.com) and then contigs were mapped using progressive Mauve alignment software to manually mitigate against false positives. A > 95% coverage and identity, plus visual confirmation using progressive Mauve [20], were used to assess relevant matches.

Sequence data from this study was submitted to the National Center for Biotechnology Information’s Sequence Read Archive (BioProject no. PRJNA872487).

## Results and discussion

### Isolate demographics

Between January 2019 and December 2020, NARA received 60 isolates harboring the OXA-48 variant, isolated from 51 patients, from hospitals and medical centers located throughout Switzerland. Fifty-five isolates were subjected to WGS and isolates were subsequently deduplicated by patient, resistance gene content and ST, leaving behind 47 non-duplicate isolates from 46 patients for further analysis. Isolates were sent from 14 cantons throughout Switzerland with Geneva (n = 12) and Zurich (n = 8) being the most represented and being the most populated areas in Switzerland. Most isolates were obtained from feces (n = 28), followed by urine (n = 9), tissue and fluid (n = 6), screening swabs (n = 2), respiratory samples (n = 1), and one isolate was obtained from an unknown sample type.

### Antibiotic resistance genes, mechanisms, and phenotypic analysis

**Fig. 1:**
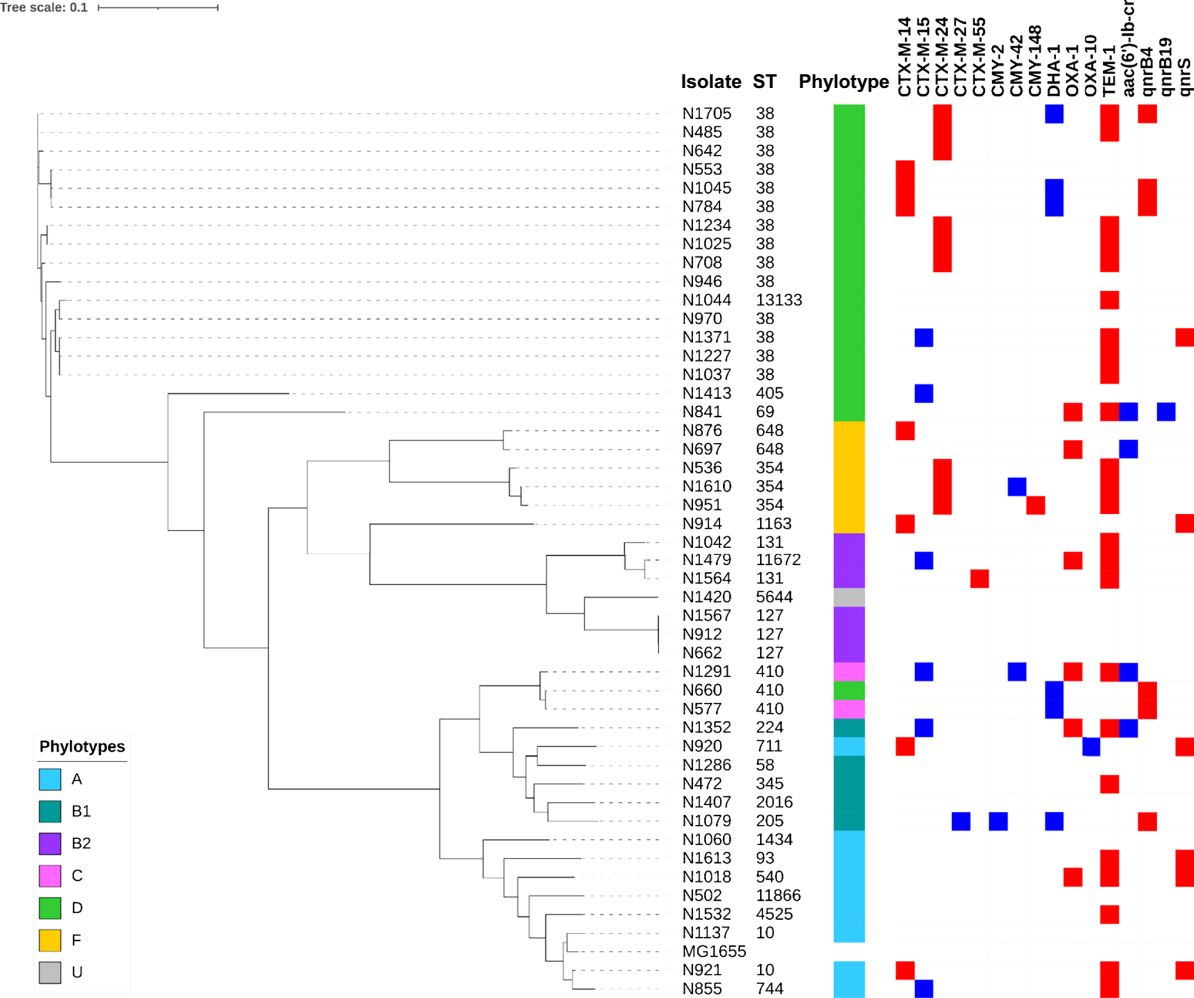
Core-genome alignment of all 47 OXA-48-producing *E. coli* strains with STs, phylotypes, ß-lactams, and plasmid-encoded quinolone resistance genes. Filled blue/red squares indicate gene presence

Susceptibility testing showed that 28 (60%), 3 (6%), and 2 (4%) isolates were resistant to the carbapenems ertapenem, meropenem, and imipenem, respectively (Table 1), according to EUCAST guidelines [14]. This result underlines that ertapenem is a useful marker for detecting OXA-48 producers [3]. Resistance to the cephalosporins cefotaxime, ceftazidime, and cefoxitin was observed in 53%, 30%, and 28% of the isolates, and 43% (20/47) were resistant to ciprofloxacin (CIP). Within the CIP-R strains, all 20 harbored mutations within the quinolone resistance-determining region (QRDR) *gyrA* and *parC* genes, and ten also harbored additional plasmid-encoded quinolone resistance genes including *qnrB4* (n = 5), *qnrS1* (n = 2), and *aac(6’)Ib-cr* (n = 3) (Fig. 1). Since OXA-48 hydrolyses carbapenems at a low level, and the expression of the corresponding gene could be quite low related to a chromosomal single-copy location, it is not unusual for OXA-48-producing *E. coli* isolates to exhibit decreased susceptibility, but not resistance, to the carbapenems in the absence of any additional resistance mechanisms (e.g., permeability loss), leading to significant problems in detecting these enzymes [6].

**Table 1:** Resistance profile of the 47 isolates according to the EUCAST guidelines. *ETP* ertapenem, *IPM* imipenem, *MEM* meropenem, *CTX* cefotaxime, *CAZ* ceftazidime, *FOX* cefoxitin, *CIP* ciprofloxacin

	Antibiotics						
	ETP	IPM	MEM	CTX	CAZ	FOX	CIP
No R/%	28/59.6	2/4.3	3/6.4	25/53.2	14/29.8	13/27.7	20/42.6

Twenty-four isolates harbored *bla*_CTX-M_-type ESBL genes (40%), corresponding to *bla*_CTX-M-24_ (n = 9), *bla*_CTX-M-14_ (n = 6), *bla*_CTX-M-15_ (n = 6), and *bla*_CTX-M-27_ (n = 1), and the remaining isolate had both *bla*_CTX-M-14_ and *bla*_CTX-M-55_. A high rate of co-association between *bla*_OXA-48_ and *bla*_CTX-M_ genes is commonly reported in many countries [3, 4, 21]. The co-occurrence of plasmid-located *ampC* cephalosporinase genes was evidenced in nine isolates, corresponding to *bla*_DHA-1_ (n = 5), *bla*_CMY-42_ (n = 2), and *bla*_CMY-142_ (n = 1), and one isolate had both *bla*_CMY-2_ and *bla*_DHA-1_. The entire antibiotic resistance gene content of all 55 sequenced isolates is described in Table S1.

Analysis of the genes encoding the main porins that can potentially interfere in the susceptibility of *E. coli* to ß-lactams showed that all isolates harbored a wild-type *ompC* gene, and only a single isolate had a truncated *ompF* gene. This relative lack of porin defects found in this collection is in accordance with the relative low levels of carbapenem resistance observed overall, keeping in mind that OmpC is known to be a critical entry point for carbapenems into *E. coli*. [22] Three isolates harbored 4 amino acid insertions within the penicillin-binding protein PBP-3, corresponding to YRIP in two ST354 isolates, and YRIN in one ST410 isolate. Such insertions have been previously shown to be associated with reduced susceptibility to PBP-3 targeting antibiotics including ceftazidime, cefepime, and aztreonam [23].

### STs and bla_OXA-48_ genetic environment

The 47 isolates comprised 25 STs, each with 1 to 3 representatives, except ST38 for which there were 14 representative isolates. The *bla*_OXA-48_ genetic location (chromosomal or plasmid) could be determined for two-thirds (32/47; 68%) of the isolates using the WGS data. Thirteen isolates of 13 different STs and obtained from 6 Swiss cantons possessed pOXA-48a plasmid or a highly related plasmid (96–100% identity) [11]. pOXA-48a has been shown to be the primary vehicle by which the *bla*_OXA-48_ gene has spread in Enterobacterales in many studies [3, 11, 24]. In seven isolates with 5 STs obtained from four cantons, the *bla*_OXA-48_ gene was encoded on a small (7,872 bp) Col156-type plasmid, whose complete DNA sequence was 100% identical that of plasmid pMTY17816_OXA48 identified in a *K. pneumoniae* isolate in Japan (GenBank NZ_AP019554.1, unpublished). These ColE-type plasmids are usually high copy number and this was confirmed by both high relative plasmid gene coverage in the WGS assemblies and read coverage in mapping analyses. Given the small size and unknown nature of this plasmid, assays were performed to determine if it was transferable and this was indeed confirmed, likely by mobilization through a larger helper conjugative plasmid since the plasmid itself does not encode any *tra* genes. Three isolates of three STs from a single canton matched 100% to a ~ 75 kb plasmid named p2-0113481141-OXA48 (Genbank CP083077, unpublished) identified in a *K. pneumoniae* isolate from Switzerland. Long read sequencing of two representative isolates and subsequent mapping with short read data identified the chromosomal location of *bla*_OXA-48_ in three ST127 isolates (obtained from two cantons), and of six ST38 isolates (from 5 cantons) which were identical to a previously published chromosomal environment (GenBank KT444704) [10]. The genetic environment for the remaining 15 isolates could not be accurately determined due to the limitations of short read sequencing for assembly; however, 6 of 15 were ST38, in which *bla*_OXA-48_ has been associated with being chromosomally encoded [10, 25].

## Conclusions

OXA-48-producing *E. coli* is increasing in incidence globally, including in Switzerland. The isolates in this study were frequently found to be associated with mechanisms conferring resistance to cephalosporins and fluoroquinolones, which are the antibiotics primarily used to treat *E. coli* infections. Low carbapenem MICs have been widely reported in OXA-48-producing *E. coli* isolates, including here, further underscoring the problematic issues related with their detection, being likely underestimated, and consequently with their dissemination [3, 4, 6]. This study shows that the wide dissemination of OXA-48-producing *E. coli* is most often either related to the successful dissemination of given strain backgrounds, particularly of ST38, and that of the well-established and highly transferable epidemic plasmid pOXA-48a which still accounts for the main process of dissemination almost 20 years after its first identification [11]. This is likely related to its frequency of transfer that is ca. tenfold higher than a “regular” resistance plasmid in Gram negatives. It is the best example of spread of a unique plasmid (and not unique clone) as a source of multidrug resistance and has not observed for any other type of carbapenemase genes (e.g., *bla*_KPC,_ *bla*_NDM_). However this study also highlights the diverse genetic environments in which the *bla*_OXA-48_ gene resides in *E. coli*, including some plasmid environments that have not yet been well described, demonstrating the fluid nature of this carbapenemase gene.

## Supplementary Information

Below is the link to the electronic supplementary material.

Supplementary file1 (DOCX 21 KB)

## Data Availability

Available upon request.
