# Supplementary material for: Molecular analysis of OXA-48-producing Escherichia coli in Switzerland from 2019 to 2020
Source: Eur J Clin Microbiol Infect Dis. 2022 Sep 14;41(11):1355–60. doi: 10.1007/s10096-022-04493-6 (PMC9556411; doi:10.1007/s10096-022-04493-6)
Supplement: Supplementary file 1 — Supplementary file1 (DOCX 21 KB) [file 10096_2022_4493_MOESM1_ESM.docx]

| **Isolate** | **ST** | **Other than *bla*_OXA-48_ Resistance Genes** |
| --- | --- | --- |
| N472 | 345 | *bla*_TEM-1_, *aadA2, sul1, tetA, dfrA12* |
| N485 | 38 | *bla*_CTX-M-24_, *bla*_TEM-1_, *aadA5, aac(3)-Iid, mphA, sul1, dfrA17* |
| N501 | 345 | *bla*_TEM-1_, *aadA2, sul1, tetA, dfrA12* |
| N502 | 11866 | None |
| N536 | 354 | *bla*_CTX-M-24_, *bla*_TEM-1_, *aadA5, mphA, sul1, tetB, dfrA17* |
| N553 | 38 | *bla*_CTX-M-14_, *strA, strB, aac(3)-Iia, sul2* |
| N577 | 410 | *bla*_DHA-1_, *mphA, qnrB4, sul1, dfrA17* |
| N642 | 38 | *bla*_CTX-M-24_, *aac(3)-Iid, aadA5, mphA, sul1, dfrA17* |
| N647 | 38 | *bla*_CTX-M-14_, *strA, strB, aac(3)-Iia, sul2* |
| N660 | 410 | *bla*_DHA-1_, *mphA, qnrB4, sul1, dfrA17* |
| N662 | 127 | None |
| N681 | 38 | *bla*_CTX-M-14_, *strA, strB, aac(3)-Iia, sul2* |
| N697 | 648 | *bla*_OXA-1_, *aac(6')-Ib-cr, aadA5, mphA, catB4, sul1, tetB, dfrA17* |
| N708 | 38 | *bla*_CTX-M-24_, *bla*_TEM-1_, *aac(3)-Iid, aadA5, mphA, sul1, dfrA17* |
| N784 | 38 | *bla*_CTX-M-14_, *bla*_DHA-1_, *strA, strB, mphA, qnrB4, sul1, sul2, dfrA17* |
| N841 | 69 | *bla*_OXA-1_, *bla*_TEM-1_, *aac(6')-Ib-cr, mphA, catB4, qnrB19, ARR-3, sul1* |
| N855 | 744 | *bla*_CTX-M-15_, *bla*_TEM-1_, *strA, strB, aph(3')-Ia, aadA5, mphA, catA1, sul1, sul2, tetB, dfrA14, dfrA17* |
| N876 | 648 | *bla*_CTX-M-14_, *aac(3)-IIa, aadA5, ermB, mphA, sul1, tetB, dfrA17* |
| N912 | 127 | None |
| N914 | 1163 | *bla*_CTX-M-14_, *strA, aph(3')-Vib, qnrS1* |
| N920 | 711 | *bla*_CTX-M-14_, *bla*_OXA-10_, *strA, ant(3'')-Ia, aph(3')-Vib, floR, cmlA1, qnrS1, ARR-3, tetA, dfrA14* |
| N921 | 10 | *bla*_CTX-M-14_, *bla*_TEM-1_, *strA, strB, aph(3')-Vib, qnrS1, tetB* |
| N946 | 38 | None |
| N951 | 354 | *bla*_CTX-M-24,_ *bla*_CMY-42_, *bla*_TEM-1_, *strA, strB, aac(3)-IId, aadA5, ermB, mphA, sul1, sul2, tetB, dfrA17* |
| N970 | 38 | None |
| N1018 | 540 | *bla*_OXA-1_, *bla*_TEM-1_, *aadA1, aph(3')-Ia, strA, strB, qnrS1, sul2, tetA, dfrA14, dfrA5* |
| N1025 | 38 | *bla*_CTX-M-24_, *bla*_TEM-1_, *aac(3)-Iid, aadA5, mphA, sul1, dfrA17* |
| N1037 | 38 | *bla*_TEM-1_, *strA, strB, aac(3)-Iid, aadA5, mphA, catA1, sul1, sul2, dfrA17* |
| N1042 | 131 | *bla*_TEM-1_, *strA, strB, aac(3)-Iid, aadA5, mphA, sul1, sul2, tetA, dfrA17* |
| N1044 | 13133 | *bla*_TEM-1_, *aadA5, mphA, sul1, dfrA17* |
| N1045 | 38 | *bla*_CTX-M-14_, *bla*_DHA-1_, *strA*, *strB,* *mphA*, *qnrB4*, *sul1*, *sul2*, *dfrA17* |
| N1060 | 1434 | *mphA* |
| N1079 | 205 | *bla*_CTX-M-27_, *bla*_CMY-2_, *bla*_DHA-1_, *aadA1, strA, strB, ermB, mphA, qnrB4, sul1, sul2, tetA, dfrA1, dfrA17* |
| N1131 | 38 | *bla*_CTX-M-14_, *bla*_DHA-1_, *strA*, *strB,* *mphA*, *qnrB4*, *sul1*, *sul2*, *dfrA17* |
| N1132 | 38 | *bla*_CTX-M-14_, *bla*_DHA-1_, *strA, strB,* *mphA*, *qnrB4*, *sul1*, *sul2*, *dfrA17* |
| N1137 | 10 | None |
| N1227 | 38 | *bla*_TEM-1_, *strA, strB, catA1, sul2, tetD,* |
| N1234 | 38 | *bla*_CTX-M-24_, *bla*_TEM-1_, *aac(3)-Iid, aadA5, mphA, sul1, dfrA17* |
| N1286 | 58 | None |
| N1291 | 410 | *bla*_CMY-42_, *bla*_CTX-M-15_, *bla*_OXA-1_, *bla*_TEM-1_, *aac(6')-Ib-cr, aadA5, fosA4, mphA, catB4, sul1, tetA, dfrA17* |
| N1352 | 224 | *bla*_CTX-M-15_, *bla*_OXA-1_, *bla*_TEM-1_, *aac(6')-Ib-cr, strA, strB, aac(3)-Iia, catB4, sul2, tetA, tetD, dfrA5, dfrA14* |
| N1371 | 38 | *bla*_CTX-M-15_, *bla*_TEM-1_, *aac(3)-Iid, aadA5, ermB, mphA, qnrS1, sul1, dfrA17* |
| N1407 | 2016 | *aadA1, ARR-3, sul1, tetA, dfrA1* |
| N1413 | 405 | *bla*_CTX-M-15_, *strA, strB, aac(3)-Iia, ermB, floR, sul2, tetA, tetB* |
| N1420 | 5644 | None |
| N1479 | 11672 | *bla*_CTX-M-15_, *bla*_OXA-1_, *bla*_TEM-1_, *aac(6')-Ib-cr* |
| N1532 | 4525 | *bla*_TEM-1_, *aadA1, strA, strB, sul1, sul2, tetA, dfrA1* |
| N1564 | 131 | *bla*_CTX-M-14_, *bla*_CTX-M-55_, *bla*_TEM-70_, *strA, strB, aac(3)-Iid, mphA, sul2, tetA* |
| N1567 | 127 | None |
| N1588 | 13133 | *bla*_TEM-1_, *aadA5, mphA, sul1, dfrA17* |
| N1610 | 354 | *bla*_CTX-M-24_, *bla*_CMY-42_, *bla*_TEM-1_, *strA, strB, aac(3)-IId, sul2, tetB* |
| N1613 | 93 | *bla*_TEM-1_, *aadA2, aac(3')-Ia, strA, strB, InuF, floR, qnrS1, sul2, tetB, dfrA12* |
| N1661 | 3014 | *bla*_CTX-M-14_, *bla*_TEM-1_, *aadA1, aadA2, aph(3')-Ia, strA, aph(3')-Vib, aac(3)-IVa, aph(4)-Ia, InuG, mefB, cmlA1, qnrS1, sul3, tetA, dfrA12* |
| N1679 | 3014 | *bla*_CTX-M-14_, *bla*_TEM-1_, *aadA1, aadA2, aph(3')-Ia, strA, aph(3')-Vib, aac(3)-IVa, aph(4)-Ia, InuG, mefB, cmlA1, qnrS1, sul3, tetA, dfrA12* |
| N1705 | 38 | *bla*_CTX-M-24_, *bla*_DHA-1_*, bla*_TEM-1_, *aadA5, aac(3)-Iid, mphA, qnrB4, dfrA17* |

**Table S1.** Sequence types and resistance gene content of all 55 OXA-48-positive *Escherichia coli* isolates.
